# Research progress of traditional Chinese medicine on the treatment of diarrhea by regulating intestinal microbiota and its metabolites based on renal-intestinal axis

**DOI:** 10.3389/fcimb.2024.1483550

**Published:** 2024-09-27

**Authors:** Tong Zhou, Yifan Zhang, Zhaoyuan Li, Chunfeng Lu, Hong Zhao

**Affiliations:** ^1^ Key Laboratory of Microecology-immune Regulatory Network and Related Diseases, School of Basic Medicine, Jiamusi University, Jiamusi, Heilongjiang, China; ^2^ College of Pharmacy, Jiamusi University, Jiamusi, Heilongjiang, China; ^3^ School of Medical, Huzhou University, Huzhou, Zhejiang, China

**Keywords:** renal-intestinal axis, traditional Chinese medicines, diarrhea, intestinal microbiota, metabolites of intestinal microbiota

## Abstract

Intestinal microbiota and its metabolites are involved in many physiological processes of the human body and play a vital role in maintaining human health. The occurrence of kidney disease can cause intestinal microbiota imbalance, resulting in diarrhea. The change of intestinal microbiota and its metabolites content can aggravate renal function injury, which has a bidirectional regulating effect. The theory of renal-intestinal axis further clarified that the impaired renal function is related to the imbalance of intestinal microorganisms, and the impaired intestinal barrier is related to the accumulation of toxin products. Because of its unique therapeutic advantages, Traditional Chinese Medicine can treat diarrhea by enhancing the growth of beneficial bacteria, inhibiting pathogenic bacteria and immune regulation, and slow down the continuous deterioration of kidney disease. This paper focuses on the relationship between intestinal microbiota and its metabolites and diarrhea, the influence of Traditional Chinese Medicine on intestinal microbiota in the treatment of diarrhea, and the role of intestinal microbiota and its metabolites in the renal-intestinal axis. It provides a theoretical basis for Traditional Chinese Medicine to regulate intestinal microbiota and its metabolites based on the renal-intestinal axis theory to treat nephrology-induced diarrhea, and also provides a new idea and method for Traitional Chinese Medicine to treat nephrology-induced diarrhea.

## 1 Introduction

As a common gastrointestinal disease, diarrhea not only affects the quality of life of patients, but also poses a certain threat to the health of the body. The intestine is an important nutrient absorption and microbial habitat of the human body, It is also the largest immune system of the human body, known as the “second brain”. The intestinal microbiota is composed of a large number of microbial communities in the intestine and has a symbiotic relationship with the host (Pushpanathan et al., 2019). They jointly maintain the homeostasis and balance of the intestinal environment by affecting the intestinal barrier function, immune response and metabolic activities, thus affecting the health of the host (Kataoka, 2016). The metabolites of intestinal microbiota are produced in the metabolic process of intestinal microbiota, which can directly or indirectly affect the physiological function of the host (Meng et al., 2020). With the deepening of research, the role of intestinal microbiota and its metabolites in maintaining human health has been paid more and more attention (Ramamurthy et al., 2022). The kidney is an important organ of the human body and the “processing center” of water and metabolites in the body. The functional state of the kidney will indirectly affect the microenvironment of the intestine. Damaged kidney function will cause intestinal microbiota imbalance. The disordered intestinal microbiota passes through the damaged intestinal mucosal barrier, causing harmful bacteria to invade and induce chronic inflammation, thus accelerating kidney damage (Kim et al., 2021; Cao et al., 2022). Renal-intestinal axis refers to the physiological and pathological mechanism of the interaction between the kidney and the intestine, and the two effect each other to form a dynamic balance system (Gong et al., 2019; Zhang et al., 2020). Traditional Chinese Medicine (TCM) has a long history of drug use in the treatment of diarrhea and shows unique advantages (Huang et al., 2023; Xue et al., 2023). Therefore, based on renal-intestinal axis theory, this paper summarizes the treatment of diarrhea by regulating the intestinal microbiota and its metabolites of TCM, and explores the role and mechanism of TCM in restoring the micro-ecological balance of the intestine, reducing the inflammatory response, and reducing the level of nephrotoxic metabolites by regulating the intestinal microbiota and its metabolites, and provides a broad application prospect for the treatment of diarrhea with TCM.

## 2 Intestinal microbiota and its metabolites and diarrhea

### 2.1 Intestinal microbiota and diarrhea

**Figure 1:**
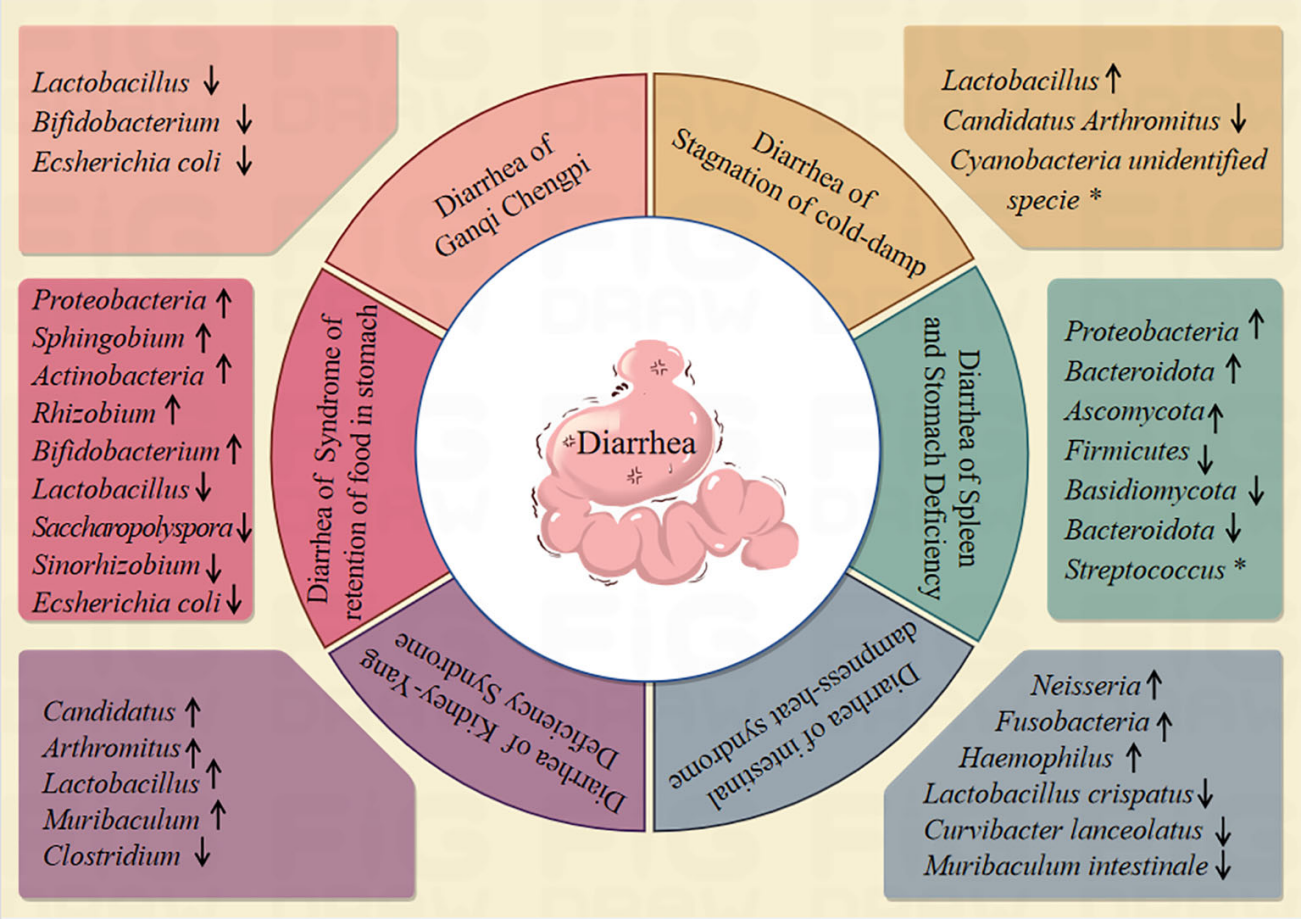
Relationship between diarrhoea and intestinal microbiota. (↑indicates an increase in intestinal microbiota abundance, ↓indicates an decrease in intestinal microbiota abundance, *indicates characteristic microbiota).

Intestinal microbiota is mainly composed of beneficial bacteria, harmful bacteria and neutral bacteria, and these microbial communities constitute a complex ecosystem in the intestine to maintain the health of the host body (Li et al., 2021a). Diarrhea, as a manifestation of intestinal dysfunction, is closely related to changes in intestinal microbiota (Shao et al., 2020; Liu et al., 2023). Diarrhea belongs to the category of TCM “diarrhea”, which can be generally divided into six syndrome types: diarrhea of intestinal dampness-heat syndrome, diarrhea of Ganqi Chengpi, diarrhea of spleen and stomach deficiency, diarrhea of stagnation of cold-damp, diarrhea of syndrome of retention of food in stomach, diarrhea of Kidney-Yang Deficiency Syndrome (Zhang et al., 2017; Li et al., 2021b). Studies have shown that diarrhea of intestinal dampness-heat syndrome model can change the structure of intestinal microbiota contents in mice, and the relative abundance of *Neisseria* were increased. The relative abundance of *Lactobacillus*, *Clostridium* and *Muribaculum* were decreased (Li et al., 2021c). An improper diet combined with high temperature and humidity environments, the abundance of *Fusobacteria* and *Haemophilus* in the model group was significantly increased (Qiao et al., 2023a). The intestinal microbiota Alpha diversity index of mice with diarrhea of Ganqi Chengpi was higher than that of normal group. The contents of, *Lactobacillus*, *Bifidobacterium* and *Ecsherichia coli* were significantly higher (Tang et al., 2020). The distribution of intestinal microbiota in patients with diarrhea, spleen and kidney Yang deficiency syndrome and liver stagnation and spleen deficiency syndrome was compared, and *Streptococcus* was the specific bacteria in the group of liver stagnation and spleen deficiency syndrome (Chao and Zhang, 2020). Intestinal microbiota of patients with diarrhea of Spleen and stomach deficiency, the relative abundance of *Firmicutes* was decreased, the relative abundance of *Proteobacteria*, *Bacteroidota* were increased (You et al., 2020). Bitter-cold purgation method with rhubarb induced to develop diarrhea of Spleen and stomach deficiency model in rats the relative abundance of *Ascomycota* was increased while the relative abundance of *Basidiomycota* and *Bacteroidota* was decreased (Xiao et al., 2023). The diarrhea of stagnation of cold-damp can change the microflora structureare. The relative abundance of *Candidatus Arthromitus* were decreased, and the relative abundance of *Lactobacillus* were increased. The *Cyanobacteria unidentified specie* were the predominant phyla of the stagnation of cold-damp diarrhea mouse model (Wu et al., 2024). In mice with diarrhea of syndrome of retention of food in stomach model, the intestinal microbiota of *Bifidobacterium*, *Lactobacillus*, *Saccharopolyspora*, *Sinorhizobium* and *Ecsherichia coli*, decreased significantly, the intestinal microbiota of *Proteobacteria*, *Sphingobium*, *Actinobacteria* and *Rhizobium* were increased (Guo et al., 2022; Zhou et al., 2022b; Zhou et al., 2023a). Diarrhea of kidney-yang deficiency syndrome can change the structure and function of intestinal microbiota in mice. The relative abundance of *Candidatus*, *Arthromitus*, *Lactobacillus*, *Muribaculum*, were increased, the relative abundance of *Clostridium* were decreased (Zhu et al., 2022; Zhou et al., 2024b). (summarized in **Figure 1**).

### 2.2 Metabolites of intestinal microbiota and diarrhea

Metabolites of Intestinal microbiota are produced by intestinal microbiota through metabolism. Metabolites of intestinal microbiota mainly include short-chain fatty acids(SCFAs), choline metabolites, lipids, vitamins, polyamines, etc (Zhang and Wang, 2024), these metabolites play an important physiological function in the intestine and can cause diarrhea. among which SCFAs is one of the important metabolites of intestinal microorganisms. Resistant starch and fiber in the colon are generated after fermentation by anaerobic bacteria, which can reduce the pH of the colon and inhibit the proliferation of pathogens, and mainly contain acetic acid, propionic acid, butyric acid, isobutyric acid, valeric acid, isovaleric acid, etc (Shu et al., 2022).

Recent studies have confirmed that SCFAs can indirectly affect the homeostasis of intestinal microbiota by regulating neurotransmitter 5-hydroxytryptamine(5-HT), dopamine and norepinephrine (Dicks, 2022). Bile acids challenge gut microbes by disrupting the stability of macromolecules, which can interfere with RNA secondary structures, cause DNA damage, and promote protein misfolding (Winston and Theriot, 2020). Lipopolysaccharide(LPS) upregulated NLRP3 levels by activating TLR4 and inducing ROS production, resulting in pyroptosis, disruption of the intestinal barrier, ultimately leading to diarrhea (Liu et al., 2024). Trimethylamine oxide (TMAO) is a harmful product of intestinal microbiota metabolism, and its production is closely related to intestinal microbiota. When intestinal microbiota changes, it will directly affect the production of TMAO and cause inflammatory response. Inflammation-related molecules can through intestinal microbiota imbalance cause diarrhea (Guo et al., 2024).

## 3 Traditional Chinese medicine for the treatment of diarrhea and metabolites of intestinal microbiota

### 3.1 Mechanism of traditional Chinese medicine in treating diarrhea

TCM has a long medicinal history in treating diarrhea. TMC believes that different intestinal microbiota are also in an interdependent but mutually restrictive dynamic balance. When this balance changes, the structure and biological characteristics of intestinal microbiota will also change, resulting in the imbalance of intestinal microbiota and thus the occurrence of diseases (Wang et al., 2024). A variety of active ingredients in TCM have bactericidal and bacteriostatic effects. While eliminating pathogenic bacteria, they regulate intestinal microbiota and restore intestinal environmental homeostasis, thereby reducing inflammatory response, repairing intestinal mucosal barrier, and treating diarrhea (He et al., 2018; Li et al., 2022b; Lai et al., 2023). The mechanisms of TMC in treating diarrhea are complex and diverse, it mainly regulates the balance of intestinal microbiota, repairs intestinal mucosal barrier, regulates intestinal motor function, and restores intestinal microbiota diversity.

#### 3.1.1 Regulation of intestinal microbiota balance

By clearing heat and detoxifying, warming the middle and dispelling cold, strengthening the spleen and stomach, TCM regulates the intestinal microecological environment, promotes the growth of beneficial bacteria and inhibits the reproduction of harmful bacteria, so as to restore the balance of intestinal microbiota (Li et al., 2022). Gegen Qinlian decoction can reverse the decrease in the richness of intestinal microbiota, significantly increase the relative abundance of SCFA-producing bacteria, and regulate intestinal microbiota (Liu et al., 2019; Li et al., 2021d). “Butuyajie” formula has the effects of regulating soil tonifying tower, clearing heat and detoxification, astringent and diarrhea, regulating the imbalance of intestinal microbiota in rats with diarrhea, increasing the abundance of *Firmicutes* and decreasing the abundance of *Bacteroidetes* and *Proteobacteria*, thus achieving the effect of treating diarrhea (Xiao et al., 2023).

#### 3.1.2 Repair of intestinal mucosal barrier

Intestinal mucosa is an important place for direct contact and communication between the internal environment and the outside world, and is the first line of defense against invasion of pathogens and harmful substances. The integrity of intestinal mucosal barrier is a prerequisite for maintaining normal intestinal function and health. Xianglian pills can restore the intestinal microbiota of mice with diarrhea, increase the expression of tight-link protein and the content of SCFAs, reduce the level of pro-inflammatory factors, alleviate intestinal mucosal damage, and improve diarrhea symptoms (Yang et al., 2021). Pingwei san can reduce inflammatory cell infiltration in the colon, promote the expression of aquaporins and tight junction markers, and play a therapeutic role in rhub-induced spleen-deficiency diarrhea in rats by protecting the intestinal barrier and regulating the imbalance of intestinal microbiota (Fan et al., 2023). TCM such as *Zingiber officinale* Rosc., *Coptis chinensis* Franch., and *Panax quinquefolius* L. and so on, also have the effect of reducing intestinal inflammation and repairing intestinal mucosal barrier (Zhou et al., 2021; Zhou et al., 2023; Kim et al., 2024).

#### 3.1.3 Regulation of intestinal motor function

The main function of gastrointestinal motility is to regulate the passage of food and its residues for optimal digestion and absorption. imbalance between the mechanisms of absorption and secretion in the intestinal tract with loss of excess fluid in the stools, and stimulates peristaltic activity in the small intestines, leading to changes in intestinal mucosa permeability to electrolytes, it will cause diarrhea (Jabri et al., 2016). TCM is often used to regulate the intestinal movement function of Qi-regulating and digestion drugs, such as Baohe pill decoction and Weichang’an pills, to alleviate the occurrence of diarrhea by regulating qi and guiding stagnation and promoting intestinal peristalsis (Wei et al., 2018; Zhou et al., 2022a).

#### 3.1.4 Restoration of intestinal microbiota diversity

The diversity of intestinal microbiota is a key factor in maintaining the homeostasis of intestinal microbial environment. It not only helps to repair intestinal microbiota structural disorders, but also promotes body health by increasing the abundance of beneficial bacteria and reducing the proportion of harmful bacteria (Xie et al., 2019; Miao et al., 2023). Chinese yam can improve diarrhea by increasing the diversity of intestinal microbiota, increasing the abundance of beneficial bacteria, improving the disorder of intestinal microbiota, and increasing the level of SCFAs (Zhang et al., 2019). *Poria cocos* Polysaccharide can improve αlpha diversity and beta diversity of intestinal microbiota mice with antibiotic-induced diarrhea, thus achieving the therapeutic effect on diarrhea (Xu et al., 2023). Qiweibaizhu powder can increase the abundance of *Actinobacteria*, *Bacteroidetes* and *Proteobacteria* in the intestinal mucosa of antibiotic-associated diarrhea mice, and restore the richness and diversity of intestinal microbiota (Hui et al., 2020).

### 3.2 The relationship between traditional Chinese medicine in the treatment of diarrhea and metabolites of intestinal microbiota

There is a close relationship between the treatment of diarrhea with TCM and the metabolites of intestinal microbiota. Mainly embodied in TCM can adjust the structure and function of intestinal microbiota, affect intestinal metabolites generated, and the amount and type of change. Through the antibacterial, anti-inflammatory and immune regulation functions of the active ingredients of TCM, they directly or indirectly act on intestinal microbiota, inhibit the growth of harmful bacteria, promote the reproduction of beneficial bacteria, and achieve the homeostatic balance of metabolites, thus achieving the purpose of treating diarrhea.

#### 3.2.1 Regulates the generation of SCFAs

Changes in the structure of intestinal microbiota can affect the production of metabolites. SCFAs are metabolites of intestinal microbiota and are important agents of interaction between host and intestinal microbiota (Parada Venegas et al., 2019; Qiao et al., 2023b; Gallardo et al., 2024). Pomegranate peel polyphenols can generate abundant SCFAs by promoting the generation of SCFAs, improving the intestinal environment and alleviating the occurrence of diarrhea (Shi et al., 2022). Shenling Baizhu powder can reduce lipid metabolism disorders, regulate intestinal microbiota imbalance, significantly increase the content of acetic acid, butyric acid and valeric acid in SCFAs, and treat diarrhea (Qiao et al., 2024). In addition, TCM such as Atractylodes macrocephala and Aucklandia lappa can improve the structure of intestinal microbiota, reduce the expression of 5-HT and butyric acid, increase the expression of the 5-hydroxytryptamine-4 receptor and 5-hydroxytryptamine transporter protein in colon tissue, and alleviate diarrhea (Li et al., 2024).

#### 3.2.2 Influence on the production of biogenic amines

Biogenic amines and other metabolites can promote intestinal peristalsis and increase intestinal permeability (Kuo et al., 2024). The heat-clearing antidote in TCM can relieve diarrhea symptoms by inhibiting the growth of harmful bacteria and reducing the production of harmful metabolites such as biological amines. TCM such as Aqueous cinnamon extract direct inhibition Both gene and protein levels of the colonic 5-HT synthetase, Tryptophan Hydroxylase 1 were also decreased in cinnamon extract treated irritable bowel syndrome(IBS) rats (Yu et al., 2023). Si-Ni-San can improve the abnormal intestinal microbiota induced by chronic restraint stress and inhibit the expression of dopamine β hydroxylase and c-fos in rat ventricles induced by chronic restraint stress. Inhibition of abnormal energy metabolism and decreased expression of occlusive hormone, The content of enterochromaffin cells, mast cells and 5-HT was inhibited (Chen et al., 2022).

#### 3.2.3 Regulate the metabolic pathway of bile acids

Bile acids, the general name of cholanates in bile, play an important role in intestinal motility, lipid digestion and bacterial growth. Bile acids can induce accelerated colon movement, improve visceral sensitivity, and ultimately lead to an increase in the content of bile acids in stool (Ticho et al., 2019; Guo et al., 2022). Some components of TCM can improve the intestinal environment by regulating the metabolic pathway of intestinal microbiota, affecting the generation and metabolism of metabolites such as bile acids. Qiwei Baizhu powder to anti-diarrheal by increased the levels of deoxycholic acid and beta-muricholicacid and decreased those of taurocholate acid, tauro-alpha-muricholic acid, and tauro-beta-muricholic acid (Xie et al., 2022). The anti-diarrhea effect of Aconite aqueous was associated with significantly increased fecal taurocholic acid, deoxycholic acid, lithocholic acid, glycochenodeoxycholic acid, dehydro-lithocholic acid, and 12-ketolithocholic acid restoring bile acids homeostasis (Zhang et al., 2023).

#### 3.2.4 Regulation of lipopolysaccharide synthesis

**Figure 2:**
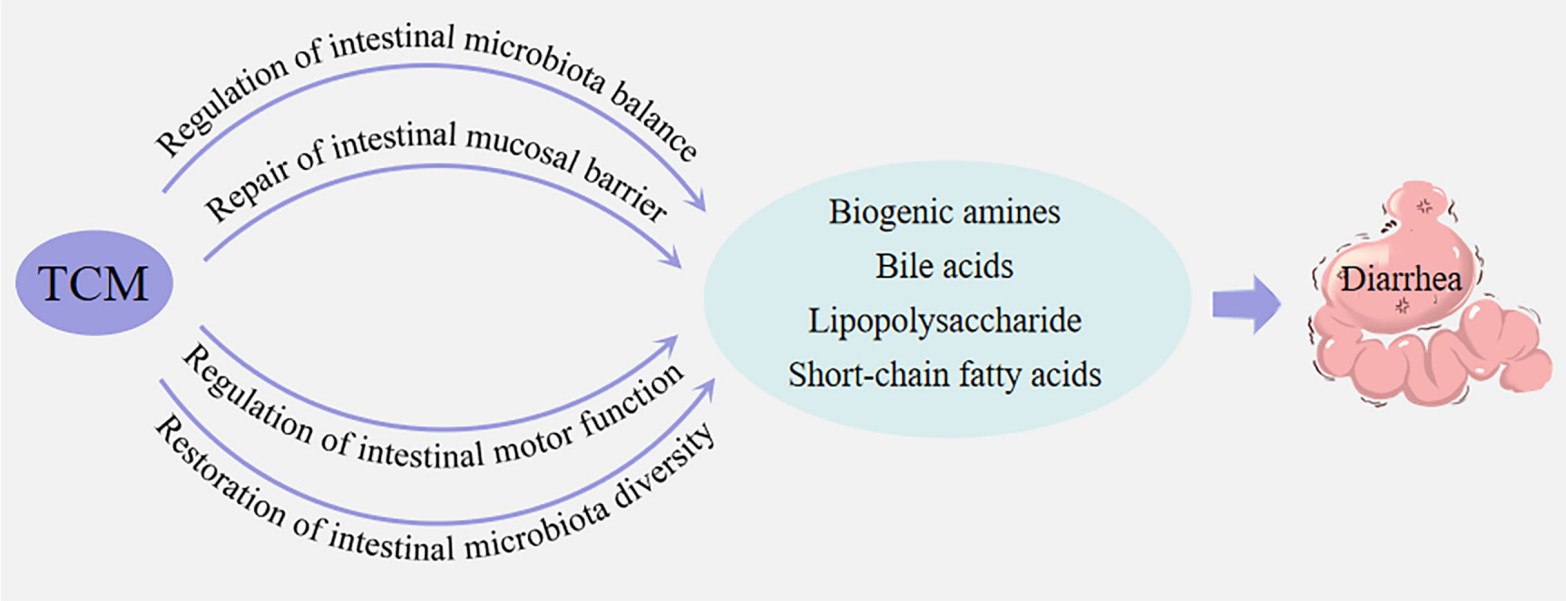
TCM treatment pathway for diarrhea.

LPS is an important component of the outer membrane of gram-negative bacteria. When it enters the blood circulation, it will cause mild inflammatory response of the body. LPS not only acts on epithelial cells, but also regulates the immune response of the body, thus maintaining the immune homeostasis of the body, and has a regulatory effect on multiple tissues of the body. The combination of *Persicaria hydropiper* (L.) can regulate the production of LPS and maintain the mucosal barrier of intestinal microbiota, thus achieving the purpose of treating diarrhea (Cheng et al., 2024). Huosha oral liquid can improve the symptoms of irritable bowel syndrome diarrhea patients and reduce the levels of serum diamine oxidase, D-lactate and endotoxin, so as to play a role in the intervention of diarrhea (Lin et al., 2021). (summarized in **Figure 2**).

## 4 The role of intestinal microbiota and its metabolites in renal-intestinal axis

### 4.1 Renal-intestinal theory

The concept of “enterorenal syndrome” was first proposed in 2011, and this theory reveals the close connection between the kidney and the intestinal in several key biological processes such as inflammation, immunity, and metabolism. It not only promoted the formation of the renal-intestinal axis theory, but also created a new therapeutic perspective to treat a variety of kidney diseases by adjusting the intestinal microbiota (Evenepoel et al., 2017). According to the renal-intestinal axis theory, the intestine is not only a key place for the digestion and absorption of nutrients, but also the largest immune organ in the human body. The intestinal immune system consists of intestinal microorganisms, epithelial cells and immune cells, and their interactions jointly maintain the defense mechanism against pathogens (Qian et al., 2016). The renal-intestinal axis theory has been thoroughly investigated recently from a variety of angles, including immunology, chemoinformatics, and molecular biology, confirming the link between the intestine and the kidney. As our understanding of the relationship between intestinal microbiota and its metabolites and kidney disease has deepened, it has become clear that both the intestinal microbiota and its metabolites are crucial for keeping the human body in a healthy state and that there is a significant correlation between the development of kidney disease and changes in the intestinal microenvironment. At present, based on the renal-intestinal axis theory, it is clear that the interaction between kidney and intestine is bidirectional, which provides a new scientific basis and treatment strategy for the diagnosis and treatment of kidney diseases (Li et al., 2023a; Xie et al., 2024a; Zhou et al., 2024a).

### 4.2 The role of intestinal microbiota and its metabolites in renal-intestinal axis

**Figure 3:**
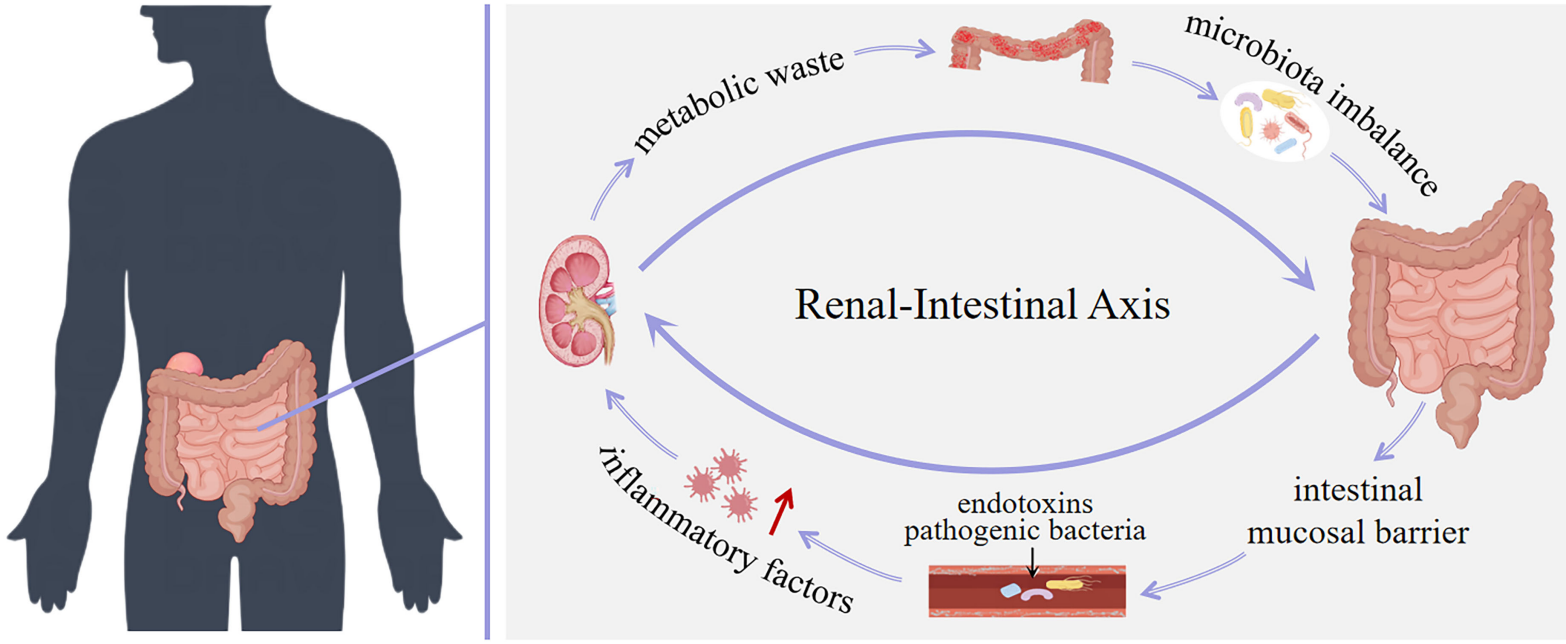
Interaction between the kidney and the intestine.

The metabolites of intestinal microbiota and play a crucial role in the renal-intestinal axis. As a bridge of information communication between the kidney and the intestine, they affect the health and functional stability between the kidney and the intestine through various mechanisms such as affecting metabolic pathways, maintaining intestinal barrier function, and regulating immune responses (Li et al., 2023; Li et al., 2024). On the one hand, the metabolic waste produced by the body in patients with kidney disease cannot be excreted in time, which leads to accumulation in blood and tissues. These harmful substances penetrate into the intestinal cavity through the mesenteric vessel wall, leading to imbalance of intestinal microbiota and its metabolites. On the other hand, the imbalance of intestinal microbiota and its metabolites destroys the intestinal mucosal barrier, damages intestinal epithelial cells, increases intestinal permeability, and intensifies the intestinal absorption of harmful substances. At the same time, pathogenic bacteria and endotoxins enter the blood circulation, making the level of inflammatory factors in the blood significantly increase, inducing systemic inflammation, aggravating the kidney burden, and aggravating the development of kidney disease. Again and again, the two formed a vicious circle. In conclusion, intestinal microbiota and its metabolites play an important role in renal-intestinal axis homeostasis (Li et al., 2022; Li et al., 2023b). (summarized in **Figure 3**).

## 5 Traditional Chinese medicine treats diarrhea caused by kidney disease by regulating intestinal microbiota and its metabolites

Because of the characteristics of multi-component, multi-target and so on, TCM has shown unique advantages in the treatment of diseases. Kidney disease can induce diarrhoea. TCM compound preparations can regulate intestinal microbiota and its metabolites through the renal-intestinal axis pathway, repair intestinal barrier damage, accelerate the excretion of toxins in the body, and slow down the further development of kidney disease (Zhou et al., 2023b; Li et al., 2024). (as shown in **Table 1**).

**Table 1:** “TCM - Nephropathy - Intestinal microbiota and its metabolites” relationship.

TCM	Disease	Molding	Object of study	Intestinal flora microbiota and its metabolites	References
Niaoduqing granules	Hypertensive nephropathy	Non-dialysis hypertensive nephropathy patients	Human	Increasing the number of bacteria producing SCFAs and repairing intestinal epithelial barrier function	(Chen, 2023)
*Scutellaria baicalensis* Georgi and *Sophora japonica* L.	Hypertensive nephropathy	Spontaneous hypertension	Rats	Decreased the ratio of *Firmicutes*/*Bacteroidetes*, increased the relative abundance of *Lactobacillus*, and reduced that of *Clostridiaceae* and improved intestinal barrier	(Guan et al., 2021)
Qing-Re-Xiao-Zheng formula	Diabetic nephropathy	High-fat diet induction combined with streptozotocin	Mice	Modulating intestinal microbiota and inhibiting inflammatory responses	(Gao et al., 2021)
Vaccarin	Diabetic nephropathy	High fat diet combined with streptozotocin	Mice	The therapeutic effect was achieved by reducing the bile acid level and the ratio of *Bacteroidetes* to *firmicutes*	(Yu et al., 2024)
Cordyceps cicadae polysaccharides	Diabetic nephropathy	High-fat diet and injected streptozotocin	Rats	Suppressing the inflammatory response and modulating intestinal microbiota dysbiosis	(Yang et al., 2020)
Resveratrol	Diabetic nephropathy	Genetic diabetic nephropathy mouse model	Mice	Improves intestinal barrier function and ameliorates intestinal permeability and inflammation	(Cai et al., 2020)
Zicuiyin decoction	Diabetic nephropathy	Diabetic	Human	Increased *Prevotellaceae* and *Lactobacillaceae* and decreased *Enterobacteriales*, *Clostridiaceae* and *Micrococcaceae*, ameliorated intestinal microbiota dysbiosis	(Liu et al., 2022)
Fufang-zhenzhu-tiaozhi formula	Diabetic nephropathy	High-fat diet and injected streptozotocin	Mice	Restored the colonic mucosal barrier elevated the content of short-chain fatty acids (propionic acid and butanoic acid) and increased the level of the SCFAs	(Lan et al., 2023)
DahuangMudan decoction	Chronic kidney disease and hyperuricemia	Unhealthy diet and lifestyle	Human	Regulate intestinal microbiota and reduce inflammatory response	(Zhang et al., 2023)
Walnut Meal polyphenol	Hyperuricemia and uric acid nephropathy	Yeast paste and potassium	Mice	Increase the relative abundance of beneficial intestinal microbiota	(Xu et al., 2024)
Curcumin	Hyperuricemia and uric acid nephropathy	Adenine and potassium oxonate	Rats	Inhibits the growth of pathogenic bacteria and increases the abundance of beneficial bacteria that produce SCFAs	(Xu et al., 2021)
Shiwuwei Rupeng pills	Hyperuricemia and uric acid nephropathy	Adenine combined with ethambutol,	Rats	Increased the richness and diversity of intestinal microbiota, regulating the structure of intestinal microbiota	(Xie et al., 2022)
Qiong-Yu-Gao	Acute Kidney Injury	Cisplatin induction	Mice	Significantly attenuated cisplatin-induced AKI and intestinal dysbiosis, altered the levels of bacterial metabolites	(Zou et al., 2022)
Emodin	Acute kidney injury	Intraperitoneal injection of gentamicin sulfate	Rats	Increased *Escherichia coli* and *Enterococcus* andregulate the imbalance of intestinal microbiota	(Sun et al., 2019)
Liangxue Huoxue decoction	Acute kidney injury	Cisplatin induction	Mice	Improve the composition of intestinal microbiota, reduce the relative abundance of *Enterococcus* and *Escherichia-Shigella*, and significantly increase the relative abundance of *Lactobacillus* and *Akkermansia*	(Zhou et al., 2023)
Chinese medicine Sishen pills	Kidney-yang deficiency type diarrhea	Adenine suspension and Folium Senna decoction	Mice	By adjusting intestinal microbiota to reduce the inflammatory response that is transmitted through the “renal-intestinal axis” as a result of elevated TMAO levels	(Xie et al., 2024b)
Chinese medicine Sishen pills	Kidney-yang deficiency type diarrhea	Adenine suspension and Folium Senna decoction	Mice	Improved the diversity and structure of intestinal mucosal microbiota	(Zhu et al., 2023)
Sisi Guben granule	Kidney-yang deficiency type diarrhea	Gavage of Senna and water avoidance stress.	Rats	Effectively reverse the disorder of intestinal bacteria in irritable bowel syndrome-diarrhea ratsto increase the abundance of probiotics, and reduce the abundance of pathogenic bacteria, promotethe absorption of metabolites of intestinal microbiota which is the SCFAs	(Xu et al., 2023)
Epimedium	Kidney-yang deficiency diarrhea	Hydrocortisone induction	Rats	Improve metabolic disorders associated with kidney yang deficiency syndrome by acting on the intestinal microbiota	(Huang et al., 2024)
Polysaccharides from Plantaginis Semen	Membranous nephropathy	Tail vein injection of cationic bovine serum albumin	Rats	Down-regulated the expression of proinflammatory factors and the content of short-chain fatty acids in renal and colon tissues, and promoted the expression of anti-inflammatory factors in regulatory cells	(Zhao et al., 2021)
Jianpi Huoluo formula	Membranous nephropathy	Patients with idiopathic model nephropathy	Human	Decrease the abundance of harmful bacteria and increase the abundance of beneficial bacteria	(Wang et al., 2024)
Piceatannol	Chronic kidney disease	Adenine suspension induction	Mice	Regulate intestinal microbiota and reduce systemic inflammation	(Li et al., 2022a)
Fucoidan	Chronic kidney disease	Adenine suspension	Mice	Regulating specific intestinal microbiota and their metabolic functions	(Lv et al., 2024)
*Panax notoginseng* saponins	Chronic kidney disease	Adenine induction	Rats	Regulation of intestinal microorganisms and inhibition of the activation of pro-inflammatory	(Xie et al., 2022)
Phytolacca Radix	Doxorubicin-induced nephropathy	Tail intravenous injection of doxorubicin hydrochloride	Rats	Improve the imbalance of intestinal microbiota	(Li et al., 2019)
Jiawei Linggui Zhugan decoction	Nephrofibrosis	Unilateral ureteral obstruction	Rats	Regulate intestinal microbiota and reduce inflammatory infiltration reduce the degree of intestinal mucosal edema, and protect intestinal barrier	(Li et al., 2021)

## 6 Summary and prospect

With the increasing attention paid to the theory of renal-intestinal axis in the treatment of nephropathy, scientific research has begun to explore the essence of renal-intestinal axis from the fields of immunology, molecular biology, chemical informatics, etc., and confirmed the multifaceted and multi-level connection between the kidney and the intestine. With the study of the interaction between intestinal microbiota and its metabolites and nephropathy induced diarrhea, it is found that the intervention of intestinal microbiota and its metabolites is a potential target for the treatment of kidney disease. Due to its unique therapeutic advantages, based on this characteristic target, TCM can achieve the purpose of treating diarrhea by regulating the balance of intestinal microbiota, changing the content of intestinal microbiota and its metabolites, and promoting the repair of intestinal mucosa, thereby slowing down the development of kidney disease. At present, the molecular mechanism of TCM treating the imbalance of intestinal microbiota and its metabolites in the renal-intestinal axis is still unclear. Therefore, it is necessary to further explore the theoretical research of renal-intestinal axis and the specific mechanisms of diarrhoea induced through kidney disease treated with TCM, so as to further guide the clinical application.
