# Corrigendum: Research progress of traditional Chinese medicine on the treatment of diarrhea by regulating intestinal microbiota and its metabolites based on renal-intestinal axis

**DOI:** 10.3389/fcimb.2024.1524374

**Published:** 2024-11-19

**Authors:** Tong Zhou, Yifan Zhang, Zhaoyuan Li, Chunfeng Lu, Hong Zhao

**Affiliations:** ^1^ Key Laboratory of Microecology-immune Regulatory Network and Related Diseases, School of Basic Medicine, Jiamusi University, Jiamusi, Heilongjiang, China; ^2^ College of Pharmacy, Jiamusi University, Jiamusi, Heilongjiang, China; ^3^ School of Medical, Huzhou University, Huzhou, Zhejiang, China

**Keywords:** renal-intestinal axis, traditional Chinese medicines, diarrhea, intestinal microbiota, metabolites of intestinal microbiota

In the published article, there was an error in affiliations. Instead of “Tong Zhou^1†^, Yifan Zhang^1†^, Zhaoyuan Li^1^, Chunfeng Lu^1,2*^ and Hong Zhao^1*^; ^1^College of Pharmacy, Jiamusi University, Jiamusi, Heilongjiang, China, ^2^School of Medical, Huzhou University, Huzhou, Zhejiang, China”, it should be “Tong Zhou^1,2†^, Yifan Zhang^2†^, Zhaoyuan Li^2^, Chunfeng Lu^1,3*^ and Hong Zhao^2*^; ^1^Key Laboratory of Microecology-immune Regulatory Network and Related Diseases, School of Basic Medicine, Jiamusi University, Jiamusi, Heilongjiang, China, ^2^College of Pharmacy, Jiamusi University, Jiamusi, Heilongjiang, China, ^3^School of Medical, Huzhou University, Huzhou, Zhejiang, China”.

The authors apologize for this error and state that this does not change the scientific conclusions of the article in any way. The original article has been updated.

